# MATI: An efficient algorithm for influence maximization in social networks

**DOI:** 10.1371/journal.pone.0206318

**Published:** 2018-11-01

**Authors:** Maria-Evgenia G. Rossi, Bowen Shi, Nikolaos Tziortziotis, Fragkiskos D. Malliaros, Christos Giatsidis, Michalis Vazirgiannis

**Affiliations:** 1 École Polytechnique, Palaiseau, France; 2 CentraleSupélec, University of Paris-Saclay and Inria Saclay, Gif-sur-Yvette, France; Universidad Rey Juan Carlos, SPAIN

## Abstract

*Influence maximization* has attracted a lot of attention due to its numerous applications, including diffusion of social movements, the spread of news, viral marketing and outbreak of diseases. The objective is to discover a group of users that are able to maximize the spread of influence across a network. The greedy algorithm gives a solution to the Influence Maximization problem while having a good approximation ratio. Nevertheless it does not scale well for large scale datasets. In this paper, we propose Matrix Influence, MATI, an efficient algorithm that can be used under both the Linear Threshold and Independent Cascade diffusion models. MATI is based on the precalculation of the influence by taking advantage of the simple paths in the node’s neighborhood. An extensive empirical analysis has been performed on multiple real-world datasets showing that MATI has competitive performance when compared to other well-known algorithms with regards to running time and expected influence spread.

## Introduction

During the last decade, online social networking sites (e.g., Facebook, Twitter, LinkedIn, Tumblr etc.) and relevant smartphone applications have caused a remarkable growth of research on social networks. This led to the development of many applications of social networks of which a rich body of studies has been classified as *the analysis of influence or information diffusion in social networks*.

Influence propagation studies have found applications in various fields. From studying human, animal or even plant epidemics [1–3] to viral marketing [4], social media analytics [5], spread of rumors [6], expert finding [7], recommendation systems [8–10], etc. A key task in order to understand information and influence diffusion is the identification of vital nodes that play a significant role in these cases. Such nodes may allow us to control the spread of an epidemic [11, 12], to predict successful scientists and scientific publications based on co-authorship and citation networks [13–15], to design influential advertisements for new products [4, 16], etc.

For example, in the case of virus propagation, such as influenza, the transmission of the disease mainly depends on the extend of contacts of the infected person to the susceptible population; thus, being able to locate and vaccinate individuals with good spreading properties can prevent from a potential outbreak of the disease, leading to efficient strategies of epidemic control. In a similar way, suppose that our goal is to promote an idea or a product in order to be adopted by a large fraction of individuals in the network. A key idea behind viral marketing is the word-of-mouth effect [17]; individuals that have already adopted the product, recommend it to their friends who in turn do the same to their own social circle, forming a cascade of recommendations [18]. The basic question here is how to target a few initial individuals (e.g., by giving them free samples of the product or explaining them the idea), that can maximize the spread of influence in the network, leading to a successful promotion campaign.

Nevertheless, locating these users in a network is not a trivial task and numerous research has been conducted to solve the problem in the area [19]. It has been of significant importance to identify these nodes that will maximize the influence and information diffusion at the end of a respective phenomenon in a network. The problem is actually split into two subtopics: i) Identification of *individual influential nodes* that have good spreading properties [20–23] and ii) Identification of *a group of nodes* that by acting all together will maximize the total spread of influence in a network [24–30], or as usually called *Influence Maximization* (IM). Indeed, the two tasks greatly differ, as finding a ranking of the nodes that by acting individually can influence a great part of the network cannot directly be used to discover the set of nodes that will—by acting at the same time—maximize the spreading of information in a graph. Of course, this is justified by the fact that putting some of the most influential spreaders together will not result in a most influential set of these spreaders, because their respective influences may be, and is usually, largely overlapped.

### Identification of a group of influential spreaders

In this work we will be studying the specific problem that concerns the identification of a group of influential spreaders. Influence maximization can formally be described as follows: given a *social network* where the relations among users are revealed, a *diffusion model* that simulates how information propagates through the network and a parameter *k*, the goal is to locate those *k* users (represented as nodes in the graph) that maximize the spread of influence.

Kempe et al. [29] formulated the problem in the aforementioned manner and proved that it is NP-hard while adopting two diffusion models borrowed from mathematical sociology: the *Linear Threshold* (LT) and the *Independent Cascade* (IC) model. According to both, at any discrete time step a user can be either active or inactive (i.e., has adopted the product or not) and the information propagates until no more users can be activated. The authors proved that the function of the influence spread (i.e., the function that gives the number of the nodes that will be active at the end of the spreading process) under both LT and IC models is monotone and submodular in respect to the number of the seed nodes that trigger the information spreading. By exploiting these properties, they presented a greedy algorithm that achieves (1 − 1/*e*) approximation ratio.

However, as the greedy algorithm repeatedly selects in every iteration the node with the maximum marginal gain by running Monte Carlo simulations, we are lead towards great performance downsides. For that reason the majority of the literature has been providing approximate rather than the exact solution.

## Related work

Following the seminal work by Kempe et al. [29], a series of algorithms have been proposed in order to: (i) reduce the number of influence spread evaluations, (ii) make batch computations of the influence spread, and (iii) design scalable heuristics towards computing the respective spread.

Leskovec et al. [30] proposed the CELF algorithm, based on a “lazy-forward” optimization scheme, that finds near-optimal solutions guaranteed to achieve at least 1/2(1 − 1/*e*) of the optimal ones by being 700 times faster than the greedy algorithm. The fact that the marginal gain of a node cannot be greater than the one achieved in previous iterations is taken into account. A table which stores every node and its marginal gain (with regards to the influence achieved by the so far selected candidates) is stored in decreasing order. Only the marginal gain of the most profitable (top) node is re-evaluated when it is needed and the table is sorted again. The node remaining at the top of the table is selected as the next seed node. Chen et al. [31] designed new algorithms that improve the greedy one and combine techniques from the aforementioned CELF algorithm towards faster greedy algorithms. Goyal et al. [27] introduced an algorithm that further optimized CELF by 35–55% in terms of running time, called CELF++. By exploiting the submodularity property of the spread function for the diffusion models (e.g., LT and IC) it avoids the unnecessary re-computations of the marginal gains.

Chen et al. [24] proposed the LDAG algorithm which is tailored for the LT model and achieves to produce results by being orders of magnitude faster than the greedy algorithm. They firstly show that the computation of influence in directed acyclic graphs (DAGs) can be done in linear time. Based on that, they construct a local DAG for every node of the network and restrict the influence of the node in this local area. After constructing the DAGs the greedy seed selection approach is applied together with an accelerated solution for updating the incremental influence spread of each node.

Jiang et al. [32] used simulated annealing to solve the IM problem and proposed the SAEDV algorithm which runs 2-3 times faster than the greedy while its accuracy is also improved. The MIA algorithm, proposed by Chen et al. [25], is a maximum influence arborescence model based on the assumption that information diffusion occurs according to the IC model. They succeed in proposing a scalable algorithm which produces results that outperform the other so far proposed heuristics by 100–120% in terms of running time. They propose i) a best-effort algorithm that estimates the upper bounds of location-aware influence spread and prunes users having small influences thus achieving an approximation ratio of (1 − 1/*e*) and ii) a topic-materialization-based algorithm that estimates the bounds of influence spread and avoids computation of the actual influence of least influential users while finally achieving an approximation of *ϵ*(1 − 1/*e*) for any for any *ϵ* ∈ (0, 1].

Jung et al. [33] proposed IRIE which incorporates fast iterative ranking algorithm (IR) with a fast influence estimation (IE) method. By avoiding to store and compute local data structures they result in compelling savings in memory usage and running time. Goyal et al. [28] proposed SimPath, an algorithm tailored for the LT model which computes the influence spread by enumerating simple paths within a small neighborhood. With the help of a parameter, a balance between running time and quality of the solution can be achieved.

Ohsaka et al. [34] developed a method that takes advantage of privileged nodes in social networks to spead up any breadth first search performed to test a node’s reachability, without loss of solution quality. Their algorithm has comparable running times with the aforementioned IRIE and SAEDV algorithms and is more robust when compared to PMIA. Tang et al. [35] designed the Two-phase Influence Maximization (TIM) algorithm which runs in near-linear time while also returning (1 − 1/*e* − 1/*ϵ*)-approximate solutions. In the first phase, a lower-bound of the maximum spread is calculated in order to derive a parameter *θ*. In the second phase, the parameter *θ* is used so that random reverse reachable (RR) sets (as defined by Borg et al. [36]) are sampled. The *k*-sized node set that covers a large number of RR sets is the final result. TIM supports the *triggering model* which is a general diffusion model incorporating both the LT and IC models. The same authors that proposed TIM, proposed the Influence Maximization via Martingales (IMM) algorithm [37]. The basis of this algorithm is a set of estimation techniques based on the *martingales* statistical tool [38]. IMM also adopts the two-phase paradigm of TIM but incorporates a drastically different parameter estimation phase and achieves to derive a lower bound of the maximum expected influence of a node-set of any size (OPT) no less than *OPT* ⋅ (1 − 1/*e*)/(1 + *ϵ*′)^2^ where *ϵ* is a tunable parameter.

Another interesting approach is the one proposed by Cohen et al. [26]. They designed a SKetch-based Influence Maximization (SKIM) algorithm which uses per-node summary structures called *combined reachability sketches* representing the node’s influence coverage [39]. They introduced *influence oracles* which can answer *influence queries* in an efficient way. This algorithm is designed based on the IC diffusion model. Goyal et al. [40] proposed a new probability model, the *credit distribution model*, which directly estimates influence spread by exploiting historical data. This makes the need for knowing the influence probabilities and making Monte Carlo simulations to compute the respective influence redundant—thus avoiding costly computations.

In this paper, we propose MATI, an efficient influence maximization algorithm under both the LT and IC diffusion models. By taking advantage of the possible paths that are created in each node’s neighborhood, we have designed an algorithm that succeeds in locating the users that can maximize the influence in a social network while also being scalable for large datasets. Specifically we take advantage of the fact that when we want to calculate the influence gain that a node will add to the influence of a group of nodes, we just need to subtract the influence of the common paths of the new node and those of our already existing seeds from this new node’s individual influence to the network. Our algorithm can be seen as an extension of the SimPath algorithm in what concerns its formulation for the LT model. To the best of our knowledge, taking advantage of the possible paths created and pre-calculating the influence gain of a node for the IC model though has not beed proposed, in the best of our knowledge, by any related work. The methods used to precalculate each node’s potential influence depends on the creation of matrices which may on one hand increase the memory consumption of the algorithm while at the same time facilitating the re-computation of the seeds in the case that some nodes and edges are deleted. In order to limit the computation of the possible paths and the respective probabilities of them being “active”, we use a pruning threshold *θ* that acts as a trade-off between the running time of the algorithm and the accuracy of the influence computation. Extensive experiments show that for both the cases of the LT and IC models, MATI performs better than the baseline methods both in terms of influence and computation time.

## Preliminaries

A social network is typically modeled as a directed graph *G* = (*V*, *E*), with each node *u* ∈ *V* to represent a user, and the edges *E* ⊆ *V* × *V* to reflect the relationships between users. An influence weight *p*_*u*,*v*_ ∈ [0, 1] is also associated with each directed edge (*u*, *v*) ∈ *E*, and represents the probability of node *u* to influence node *v*. Let us denote as $T\left(u\right)=\left\{\tau_{1},\tau_{2},\ldots ,\tau_{M}\right\}$ the set of all possible unique paths (*M* in total) that exist in the graph starting from node *u*, with *τ*_*i*_ ⊄ *τ*_*j*_, ∀*j* ∈ *M* and *j* ≠ *i*. Roughly speaking, any path $\tau_{i}\in T\left(u\right)$ cannot be presented as a subpath of any other path $\tau_{j}\in T\left(u\right),j\neq i$ presented in the same set of paths. It should be also noticed that our paths are not allowed to contain cycles. For all the above reasons, we use the Depth-first search (DFS) algorithm for the generation of the paths that start from node *u*, with the node *u* to be the root of the *tree*. Each path $\tau_{i}\in T\left(u\right)$ consists of a sequence of nodes *τ*_*i*_ = {*n*_*i*1_, *n*_*i*2_, …, *n*_*iN*_}, where *N* is the length of the path. *M* and *N* can obviously be different for every node *u* and every path *τ*_*i*_, respectively, but they are defined as such for the sake of the simplicity of the model.

Let $p_{\ell ,\ell +1}^{\tau_{i}}$, 1 ≤ *ℓ* ≤ *N* − 1, represent the influence weight (probability) between two successive nodes (*n*_*iℓ*_ and *n*_*i*(*ℓ*+1)_) in path *τ*_*i*_. Then, $F\left(\tau_{i}\right)=\left\{f_{i1},f_{i2},\ldots ,f_{iN}\right\}$ represents the probabilities for every subpath in *τ*_*i*_ starting from node *u* to be *a*ctive (i.e., a path is considered active if each one of its edges is active). The *f*_*ij*_ corresponds to the probability that the subpath {*n*_*i*1_, *n*_*i*2_, …, *n*_*ij*_} is active, and is defined as follows:

$$\begin{array}{c} f_{ij}=\{\begin{array}{cc} \prod_{\ell =1}^{j-1}p_{\ell ,\ell +1}^{\tau_{i}} & \text{if}\;j>1,\\ 1 & \text{otherwise}. \end{array} \end{array}$$ (1)

Let us now define as Ψ(*u*, *v*) = {*ψ*_1_, *ψ*_2_, …, *ψ*_*L*_} the set of all possible (unique) paths from a node *u* to a node *v*. *ψ*_*i*_ represents each possible path and *L* is the number of all possible paths between nodes *u* and *v*. Each path *ψ*_*i*_ consists of a sequence of nodes *ψ*_*i*_ = {*n*_*i*1_, *n*_*i*2_, …, *n*_*iN*_} where *N* represents again the number of nodes of path *ψ*_*i*_. Obviously *L* ≤ *M* and again *L* and *N* can be different for every set of paths between two nodes and every path *ψ*_*i*_, respectively. We can now respectively define as Φ(*ψ*_*i*_) = {*ϕ*_*i*1_, *ϕ*_*i*2_, …, *ϕ*_*iN*_} the probability for every path *ψ*_*i*_ between two nodes *u* and *v* which is calculated in the same way as *f*_*ij*_ (see Eq (1)).

**Fig 1:**
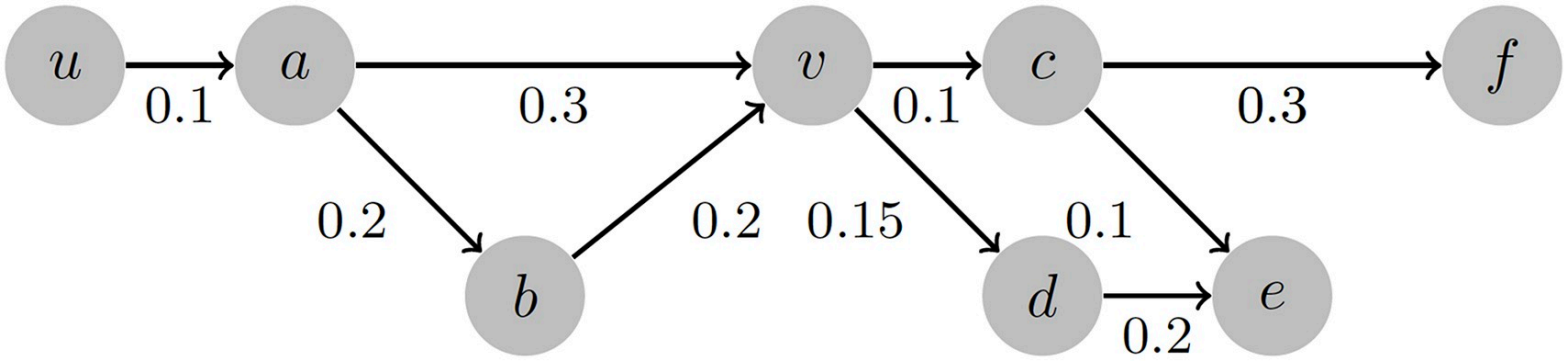
Example graph.

In this point, we will present the above notations by using a toy example. For this purpose, we consider the graph illustrated in Fig 1 that consists of |*V*| = 8 nodes and |*E*| = 9 edges. The set of paths starting from node *u* is defined as $T\left(u\right)=\left\{\tau_{1},\tau_{2},\tau_{3},\tau_{4},\tau_{5},\tau_{6}\right\}$, where:

$$\begin{array}{cc} \tau_{1} & =\{u,a,v,c,f\},\;N=5\\ \tau_{2} & =\{u,a,v,c,e\},\;N=5\\ \tau_{3} & =\{u,a,v,d,e\},\;N=5\\ \tau_{4} & =\{u,a,b,v,c,f\},\;N=6\\ \tau_{5} & =\{u,a,b,v,c,e\},\;N=6\\ \tau_{6} & =\{u,a,b,v,d,e\},\;N=6. \end{array}$$

Therefore, the probability path for *τ*_1_ is defined as: $F\left(\tau_{1}\right)=\left\{f_{11},f_{12},f_{13},f_{14},f_{15}\right\}$, where *f*_11_ = 1, *f*_12_ = 0.1, *f*_13_ = 0.03, *f*_14_ = 0.003 and *f*_15_ = 0.0009.

In the same way, the set of all possible paths from node *u* to node *v* is defined as Ψ(*u*, *v*) = {*ψ*_1_, *ψ*_2_} with *L* = 2 and the different paths between the two nodes being the following:

$$\begin{array}{cc} \psi_{1} & =\{u,a,v\},\;N=3\\ \psi_{2} & =\{u,a,b,v\},\;N=4. \end{array}$$

Then, the probability path for *ψ*_1_ is defined as: Φ(*ψ*_1_) = {*ϕ*_11_, *ϕ*_12_, *ϕ*_13_} with *ϕ*_11_ = 1, *ϕ*_12_ = 1 * 0.1 = 0.1 and *ϕ*_13_ = 1 * 0.1 * 0.3 = 0.03. Similarly, Φ(*ψ*_2_) = {*ϕ*_21_, *ϕ*_22_, *ϕ*_23_, *ϕ*_24_} with *ϕ*_21_ = 1, *ϕ*_22_ = 1 * 0.1 = 0.1, *ϕ*_23_ = 1 * 0.1 * 0.2 = 0.02 and *ϕ*_24_ = 1 * 0.1 * 0.2 * 0.2 = 0.004.

All the notations used throughout the paper are summarized in Table 1.

**Table 1:** List of symbols used in the paper.

Notation	Description
*p*_*u*,*v*_	Influence weight on directed edge (*u*, *v*)
*σ*(*S*)	Influence of a set of nodes *S* to the graph
A(u,v)	Influence of node *u* to node *v*
Ω(*u*, *v*)	Forward cumulative influence of node *u* to node *v*
$T\left(u\right)=\left\{\tau_{1},\tau_{2},\ldots ,\tau_{M}\right\}$	Set of all possible paths starting from node *u*
*τ*_*i*_ = {*n*_*i*1_, *n*_*i*2_, …, *n*_*iN*_}	Path consisting of *N* nodes starting from node *u*
$F\left(\tau_{i}\right)=\left\{f_{i1},f_{i2},\ldots ,f_{iN}\right\}$	Cumulative probability path for path *τ*_*i*_
$p_{\ell ,\ell +1}^{\tau }$	Influence weight between successive nodes in *τ*
Ψ(*u*, *v*) = {*ψ*_1_, *ψ*_2_, …, *ψ*_*L*_}	Set of all possible paths between nodes *u* and *v*
*ψ*_*i*_ = {*n*_*i*1_, *n*_*i*2_, …, *n*_*iN*_}	Path between nodes *u* and *v*
Φ(*ψ*_*i*_) = {*ϕ*_*i*1_, *ϕ*_*i*2_, …, *ϕ*_*iN*_}	Cumulative probability path for path *ψ*_*i*_

## The Influence Maximization (IM) problem

In this Section we present the problem of *Influence Maximization (IM)* as well as a description of the Linear Threshold (LT) and Independent Cascade (IC) diffusion models to which our algorithm is based.

According to Kempe et al. [29] and the spreading models they consider, the nodes are categorized in two states: the *active* and the *inactive* state. Initially a set of active nodes *S* is considered that trigger a spreading process that continues until no further activations are possible. The problem of influence maximization (IM) is to discover the called *seed set* *S* of size *k* ≪ |*V*| (*k* represents our budget) that maximizes the expected number of active nodes in the end of the process, defined as *σ*(*S*).

With the IM problem being NP-hard [29], the majority of the literature provides approximate rather than the exact solutions. The most common approximate algorithms would be: i) *heuristic* and ii) *greedy* algorithms. An example of a heuristic algorithm would be to rank all the nodes according to a centrality measure and select the *k* top-ranked nodes.

The earliest greedy algorithm was provided by Kempe et al. [29], that approximates the optimal set within a factor of (1 − 1/*e*). Initially, greedy algorithm assumes that the seed set is empty, *S* = ∅. Then at each round, it adds to the seed set the node with the maximum marginal gain. This procedure is repeated until the budget *k* is reached (|*S*| = *k*). Since the exact calculation of marginal gain is analytically intractable, it is approximated by executing multiple Monte Carlo (MC) simulation of the spreading process. Typically the number of simulations is set equal to 10.000. This constitutes a heavy burden to the greedy algorithm, making it not scalable to very large graphs.

### Diffusion models

To simulate the process of information propagation in the network different diffusion models can be used. Next, we describe two of the most widely applied models, namely the LT and IC models. As we will present later on, the proposed algorithm is designed to deal with both of those models—contrary to most of the state-of-the-art algorithms which have been designed for one of the two models.

#### Linear Threshold (LT) model

**Fig 2:**
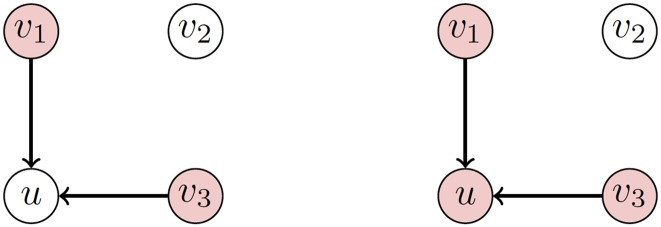
Illustration of the Linear Threshold model. The white colored nodes are in an inactive state whereas the pink colored nodes are in an active state. Node *u* will be activated if *p*_*v*_1_,*u*_+*p*_*v*_3_,*u*_ ≥ *θ*_*u*_.

In this model, a node *u* is influenced by each neighbor *v* according to a weight *p*_*u*,*v*_. The value of this weight is such that the sum of all the weights towards all neighbors of *v* is less or equal to 1. Each node *u* chooses a threshold *θ*_*u*_ uniformly at random from the interval [0, 1] which represents the weighted fraction of *u*’s neighbors that must become active in order for *u* to become active (an example is provided in Fig 2). Given a random choice of thresholds and an initial set of active nodes (with all other nodes being inactive), the diffusion process unfolds deterministically in discrete steps. A node *u* can be activated when the total cumulative weight of its active neighbors is at least *θ*_*u*_.

#### Independent Cascade (IC) model

In the IC model, when a node *u* first becomes active in timestep *t*, it is given a single chance to activate each neighbor *v*—which is currently inactive—and succeeds with a probability *p*_*u*,*v*_. If *u* succeeds, then *v* will become active in the next timestep. If *u* does not succeed, it cannot further attempt to activate *v* in future timesteps. The process continues as long as node activations are possible.

## MATrix Influence (MATI) algorithm

In this section we introduce the proposed MATI algorithm, under both the LT and IC models. Based on our previous discussion for calculating influence in social networks (Section Preliminaries), we describe in detail how we compute the influence and we provide the algorithms proposed. Similar to the case of the greedy algorithm [29], at each round of MATI, the node with the largest marginal influence estimate is chosen as the next candidate. The novelty of our algorithm lies on the fact that, by having pre-calculated all possible paths between nodes along with the respective influences of the nodes -acting individually- in the network, we are able to efficiently compute the marginal gain of adding a candidate node.

### Influence computation under the LT model

Kempe et al. [29] have shown the equivalence of the Linear Threshold model to the *live-edge model*. According to this model, a node *u* ∈ *V* chooses just one of its incoming edges with probability *p*_*u*,*v*_. If an edge is selected, it is considered *live*, otherwise *blocked*. We can deduce from the above that the nodes expected to be activated by a seed set *S* is the expected number of nodes that can be reached from *S* over all possible worlds (“All possible worlds” refers to all the different scenarios where different paths are considered live in the specific graph.). As it has been shown by Goyal et al. [28], the expected spread of seed set *S* can be calculated as follows:

$$\begin{array}{c} \sigma \left(S\right)=\sum_{v\in V}\sum_{X}\mathrm{Pr}\left[X\right]I\left(S,v,X\right)=\sum_{v\in V}A\left(S,v\right), \end{array}$$ (2)

where *X* is a possible *live-edge* graph, Pr[*X*] is the sampling probability of graph *X*, *I*(*S*, *v*, *X*) is an indicator function which equals to 1 if there exists a live path in *X* from *S* to *v* and 0 otherwise, and A(S,v) is the probability the single node *v* to be activated (influenced) by *S*. In the special case of a single node *u*, its expected spread to a node *v* (*u* ≠ *v*) is defined as:

$$\begin{array}{cc} A(u,v) & =\sum_{\psi_{i}\in \Psi \left(u,v\right)}\mathrm{Pr}\left[\psi_{i}\right]\\ & =\sum_{\psi_{i}\in \Psi \left(u,v\right)}\prod_{\ell =1}^{N-1}p_{\ell ,\ell +1}^{\psi_{i}}\\ & =\sum_{\psi_{i}\in \Psi \left(u,v\right)}\phi_{iN}=\sum_{i=1}^{L}\phi_{iN}, \end{array}$$ (3)

where Pr[*ψ*_*i*_] is the probability of path *ψ*_*i*_ being *live* and Ψ(*u*, *v*) is the set of all possible paths between nodes *u* and *v*. It becomes apparent that the influence of a node *u* to itself is equal to 1 (i.e., A(u,u)=1). According to the above, the influence of node *u* to node *v* in our example graph (see Fig 1 in Section Preliminaries) is equal to:

$$\begin{array}{c} A\left(u,v\right)=\sum_{i=1}^{2}\phi_{iN}={\left[\phi_{13}\right]}_{\psi_{1}}+{\left[\phi_{24}\right]}_{\psi_{2}}=0.034. \end{array}$$

We can now define the expected total influence spread of a single node *u* to the network:

$$\begin{array}{c} \sigma \left(u\right)=\sum_{v\in V}A\left(u,v\right)\approx \sum_{v\in I(u)}A\left(u,v\right), \end{array}$$ (4)

where *I*(*u*) represents the nodes in the graph that can be influenced by node *u* depending on a threshold *θ* which is set in order to limit the calculations of probability and cumulative probability paths. Roughly speaking, a node *v* belongs to set *I*(*u*), *iff* *A*(*u*, *v*) ≥ *θ*. Therefore, the lower the parameter value *θ* is, the higher the accuracy that can be achieved.

The *forward cumulative influence* Ω(*u*, *v*) is another quantity of interest, that corresponds to the influence of node *u* to *v* and of node *u* to the nodes that can be found right after node *v* in the paths T(u) of node *u*. Algorithms 2 and 3 show how Ω is calculated.

Practically each Ω(*u*, *v*) element is calculated as the sum of i) the probabilities that all unique paths between nodes *u* and *v* are live and ii) the probabilities that all unique paths to nodes visited after node *v* while performing a Depth-first search starting from node *u* are live. In our example graph (see Fig 1 in Section Preliminaries) the aforementioned paths are the following: {u,a,v}, {u,a,b,v}, {u,a,v,c}, {u,a,v,c,f}, {u,a,v,c,e}, {u,a,v,d}, {u,a,v,d,e}, {u,a,b,v,c}, {u,a,b,v,c,f}, {u,a,b,v,c,e}, {u,a,b,v,d}, {u,a,b,v,d,e}. According to the above, the cumulative influence of node *u* to node *v* in our example graph is equal to: Ω(*u*, *v*) = 0.03 + 0.004 + 0.003 + 0.0009 + 0.0003 + 0.0045 + 0.0009 + 0.0004 + 0.00012 + 0.00004 + 0.0006 + 0.00012 = 0.04488.

Revisiting the case of a set of nodes, Goyal et al. [28] showed that the spread of a set *S* of nodes is the sum of the spread of each individual node *u* ∈ *S* on the subgraphs induced by the set *V* − *S* + *u*:

$$\begin{array}{c} \sigma \left(S\right)=\sum_{u\in S}\sigma^{V-S+u}\left(u\right), \end{array}$$ (5)

where *σ*^*V*−*S*+*u*^(*u*) denotes the total influence of *u* in the subgraph induced by *V* − *S* + *u*. Similar to [28], we write *V* − *S* to denote the difference of sets *V* and *S*, *V* \ *S*, and *V* − *S* + *u* to denote ((*V* \ *S*) ∪ {*u*}).

By taking advantage of the A(u,v) (Eq 5) and Ω(*u*, *v*) definitions, we get the following key result that helps towards the calculation of the influence gain after the addition of a node *x* to a set of nodes *S*. This result constitutes the basis of the proposed MATI algorithm under the LT diffusion model.

**Theorem 1**. *Under the LT model, to calculate the influence after adding a node x to a set of nodes S, one has to subtract from the sum of the individual spread of S and x, the forward cumulative influence* Ω *of all the nodes that belong to set S, which contain node x in paths connecting the latter to nodes in set S. That is*,

$$\begin{array}{c} \sigma \left(S\cup \left\{x\right\}\right)=\sigma \left(S\right)+\sigma \left(x\right)-\sum_{y\in S}\Omega \left(x,y\right)-\sum_{y\in S}\Omega \left(y,x\right). \end{array}$$ (6)

*Proof*.

$$\begin{array}{cc} \sigma (S\cup \{x\}) & \overset{\left(1\right)}{=}\sum_{u\in S+x}\sigma^{V-S-x+u}\left(u\right)\\ & \overset{\left(2\right)}{=}\sigma^{V-S}\left(x\right)+\sum_{u\in S}\sigma^{V-S-x+u}\left(u\right)\\ & \overset{\left(3\right)}{=}\sigma^{V-S}\left(x\right)+\sigma^{V-x}\left(S\right)\\ & \overset{\left(4\right)}{=}\sigma \left(x\right)+\sigma \left(S\right)-\sum_{y\in S}\Omega \left(x,y\right)-\sum_{y\in S}\Omega \left(y,x\right). \end{array}$$

**Fig 3:**
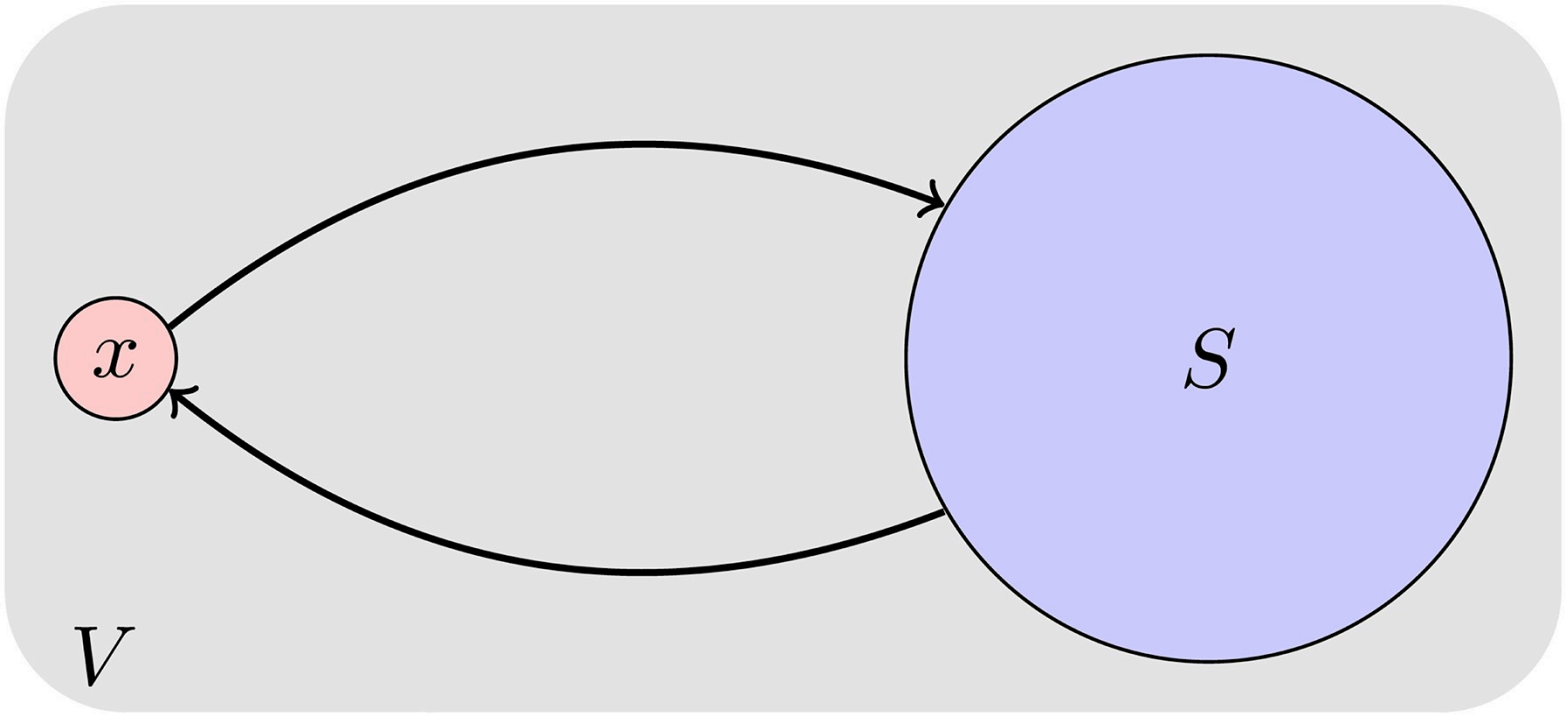
Illustration of Theorem 1.

Equality (1) is a direct application of Eq 5 (see [28] for its proof). Equalities (2) and (3) can easily be verified by making simple calculations. Finally, equality (4) comes from set theory (see Fig 3 for an illustration). Roughly speaking, equality (4) expresses that the influence gain after adding node *x* to a set *S*, is equal to the summation of the influence gain of *x* and *S* independently, subtracting from this value the nodes that can be influenced by *x* or *S*, through paths that pass via nodes on set *S* or node *x*, respectively. For instance, if both *x* and *S* influence nodes that do not cross each other, we get that *σ*(*S* ∪ {*x*}) = *σ*(*x*) + *σ*(*S*).

Algorithms 1 to 4 show the complete structure of MATI algorithm under the LT model. Routine CalcStatsLT (Algorithm 2) computes A and Ω, and routine CalcInf (Algorithm 4) returns the influence of all nodes *v* ∈ *V*, as was described in this section. We use a CELF queue which is a queue storing the nodes’ marginal gains in decreasing order, as in [27]. At each iteration, we add the top node of the CELF queue at the seed set, until the budget *k* is reached (see Algorithm 1). The influence gain of every node to be selected is calculated by subtracting the respective influence for which the candidates selected so far are responsible for, through common paths as shown in Theorem 1.

**Algorithm 1** MatiLT

1: **Input**: *G* = (*V*, *E*), *k*          ⊳ *k*: budget (number of seed nodes)

2: **Initialize**: *S* = ∅

3: A,Ω=CalcStatsLT(G)

4: Q=CalcInf(A,V)

5: **for** *i* = 1 to *k* **do**

6:  *s*, *σ*(*s*) = *Q*.*top*()

7:  *S* = *S* ∪ *s*

8:  *U* = *V*\*S*

9:  **for each** *u* ∈ *U* **do**

10:   *σ*(*u*) = *Q*(*u*)

11:   **for each** *v* ∈ *S* **do**

12:    *σ*(*u*) −= Ω(*v*, *u*)

13:    *σ*(*u*) −= Ω(*u*, *v*)

14:   **end for**

15:   *Q*.*add*((*u*, *σ*(*u*)))

16:  **end for**

17: **end for**

18: **return** *S*

**Algorithm 2** CalcStatsLT

1: **Input**: *G* = (*V*, *E*)

2: **Initialize**: A,Ω=0

3: **for each** *u* ∈ *V* **do**

4:  *A*(*u*, *u*) = 1

5:  *A*(*u*,:), Ω(*u*,:),_ = *DFStatistics*(*G*, *u*, 1, *A*(*u*,:), Ω(*u*,:))

6: **end for**

7: **return** A,Ω

**Algorithm 3** DFStatistics

1: **Input**: *G* = (*V*, *E*), *u*, *p*_*u*_, *A*_*r*_, Ω_*r*_

2: **Initialize**: Ω_*temp*_ = *p*_*u*_

3: **for each** *w* ∈ *Neighbors*(*u*) **do**

4:  *p*_*w*_ = *p*_*u*,*w*_ * *p*_*u*_

5:  *A*_*r*_(*w*) += *p*_*w*_

6:  $A_{r},\Omega_{r},\Omega_{temp}^{\prime }=DFStatistics\left(G,w,p_{w},A_{r},\Omega_{r}\right)$

7:  $\Omega_{temp}+=\Omega_{temp}^{\prime }$

8: **end for**

9: Ω_*r*_(*u*) += Ω_*temp*_

10: **return** *A*_*r*_, Ω_*r*_, Ω_*temp*_

**Algorithm 4** CalcInf

1: **Input**: A,V

2: **Initialize**: *Q* = ∅; *σ*(*u*) = 0, ∀_*u*_ ∈ *V*

3: **for each** *u* ∈ *V* **do**

4:  **for each** *v* ∈ *I*(*u*) **do**             ⊳ *I*(*u*): nodes influenced by *u*

5:   σ(u)+=A(u,v)

6:  **end for**

7:  *Q*.*add*((*u*, *σ*(*u*)))

8: **end for**

9: **return** *Q*

### Influence computation under the IC model

In the IC diffusion model, the activation probability of a node to another one in a path can be calculated by multiplying the influence weights $p_{\ell ,\ell +1}^{\tau }$ leading to it in path *ψ*_*i*_. That is, in a path *ψ*_*i*_ = {*n*_*i*1_ = *u*, *n*_*i*2_, …, *n*_*iN*_ = *v*}, the influence of node *u* to node *v* can be calculated as follows:

$$\begin{array}{c} A_{\psi_{i}}\left(u,v\right)=\prod_{\ell =1}^{N-1}p_{\ell ,\ell +1}^{\psi_{i}}=\phi_{iN}. \end{array}$$

The total activation probability of node *v* from node *u*, while taking into consideration the *L* different (unique) paths Ψ(*u*, *v*) = {*ψ*_1_, *ψ*_2_, …, *ψ*_*L*_} that lead from *u* to *v*, can be computed as:

$$\begin{array}{c} A\left(u,v\right)=1-\prod_{\psi_{i}\in \Psi \left(u,v\right)}\left(1-\sigma_{\psi_{i}}\left(u,v\right)\right)=1-\prod_{i=1}^{L}\left(1-\phi_{iN}\right), \end{array}$$

where $\prod_{i=1}^{L}\left(1-\phi_{iN}\right)$ is equal to the probability that node *u* does not influence *v* (i.e., none of the paths from *u* to *v* is active).

In the case of the IC model, Ω(*u*, *v*) cannot be calculated only according to the influence weights *p*_*k*,*k*+1_ in a specific path. In fact, the calculation of the influence in the case of the addition of a node *u* will change the so far calculated influence of a set of seed nodes *S*. Therefore, we use the following heuristics to compute the additional influence of node *u*, i.e., *σ*(*S* ∪ {*u*}).

#### *σ*(*S* + *u*) computation

1. For every path originating from node *u* (i.e., T(u)) or a node belonging to seed set *S*, we keep the subpaths before falling into a node belonging to *S* ∪ {*u*}.
2. The *σ*(*S* + *u*) is equal to the sum of the influence probabilies that correspond to each of these subpaths.

**Algorithm 5** MatiIC

1: **Input**: *G* = (*V*, *E*), *k*          ⊳ *k*: budget (number of seed nodes)

2: **Initialize**: *S* = ∅, *σ*(*S*) = 0

3: A=CalcStatsIC(G)

4: Q=CalcInf(A,V)

5: **for** *i* = 1 to *k* **do**

6:  *s*, *σ*(*s*) = *Q*.*top*()

7:  *S* = *S* ∪ *s*

8:  *U* = *V*\*S*

9:  *σ*(*S*) = *σ*(*S*) + *σ*(*s*)

10:  **for each** *u* ∈ *U* **do**

11:   *σ*(*S* ∪ {*u*}) = |*S* ∪ {*u*}|

12:   *σ*(*S* ∪ {*u*}) += AdditiveInf(T(u), F(u), *S*)

13:   *σ*(*S* ∪ {*u*}) += AdditiveInf(T(S), F(S), *S* ∪ {*u*})

14:   *Q*.*add*((*u*, *σ*(*S* ∪ {*u*}) − *σ*(*S*)))             ⊳ Order is maintained

15:  **end for**

16: **end for**

17: **return** *S*

**Algorithm 6** CalcStatsIC

1: **Input**: *G* = (*V*, *E*)

2: **Initiaze**: A=0

3: **for each** *u* ∈ *V* **do**

4:  Generate T(u) and $F(\tau_{i})$, ∀ *τ*_*i*_ using DFS

5:  **for each** *v* ∈ *V* **do**

6:   *pr* = 1

7:   Generate Ψ(*u*, *v*) and Φ(*ψ*_*i*_), ∀*ψ*_*i*_ (based on T(u))

8:   **for each** *ψ*_*i*_ ∈ Ψ(*u*, *v*) **do**

9:    *pr* = *pr* * (1 − *ϕ*_*ij*_)

10:   **end for**

11:   A(u,v)=1−pr

12:  **end for**

13: **end for**

14: **return** A

**Algorithm 7** AdditiveInf

1: **Input**: T,F,S          ⊳ T: set of paths, *S*: set of nodes

2: **Initialize**: *i* = 0; *inf* = 0

3: **for each** τ∈T **do**

4:  *i* = *i* + 1

5:  **for each** *u* ∈ *τ* **do**

6:   *j* ← *index*(*u*)

7:   **if** *j* ⩵ 1 **then**

8:    **continue**

9:   **else if** *u* ∉ *S* **then**

10:    *inf* += *f*_*ij*_

11:   **else**

12:    **break**

13:   **end if**

14:  **end for**

15: **end for**

16: **return** *inf*

Algorithm 5 shows the structure of the MATI algorithm under the IC model. Initially, routines CalcStatsIC (Algorithm 6) and CalcInf (Algorithm 4) are called to compute A and the contents of CELF queue *Q*, respectively. While calculating the marginal influence for every candidate node *u*, routine AdditiveInf (Algorithm 7) computes the additional influence as previously described (see *σ*(*S* ∪ {*u*}) computation).

## Experiments

We have conducted experiments in real-world datasets in order to evaluate the performance of the MATI algorithm and compare it to state-of-the-art influence maximization algorithms on the quality of results and efficiency. The algorithm has been implemented in Python and all experiments are run on a Linux machine with a 3.00GHz CPU Intel Xeon CPU and 64GB memory.

### Datasets

We have used four publicly available graph datasets [41] whose statistics are summarized in Table 2. To generate influence weights on all edges, we adopt the classical uniform method by [29, 42]. More precisely, we set the weight of every incoming edge of a node *v* to be equal to $\frac{1}{d_{v}}$, where *d*_*v*_ is the in-degree of node *v*. It has to be noted that the datasets were transformed from an undirected format to a directed one by simply assuming that if an edge between two nodes exists the one can influence the other. Thus, the number of the edges used in the experiments is twice the one that is reported.

**Table 2:** Properties of the real-world graphs used.

Dataset	NetHEPT	WikiVote	Epinions	Email-EuAll
# Nodes	15K	7K	75K	225K
# Edges	62K	103K	405K	341K

- NetHEPT: This is a collaboration network taken from the “High Energy Physics (Theory)” section of http://arxiv.org, with nodes representing authors and edges capturing co-authorship relationships. Here, a user publishing a paper is considered as an action.
- WikiVote: This network has been created after extracting all administrator elections and vote history data from Wikipedia, the free encyclopedia written by peers all around the world. It contains data from 2, 794 elections with 103, 663 total votes and 7, 066 users dated from the inception of Wikipedia until January 2008.
- Epinions: This is a who-trust-whom online social network of the general consumer review site *Epinions* (*epinions.com*). Each member decides whether to trust or not the other members. From the trust relationships that are created together with the review ratings provided by the users, it is determined which reviews to be shown to each user.
- Email-EuAll: This network has been generated using email data from a large European research institution for a period from October 2003 to May 2005 (18 months). Given a set of email messages, each node corresponds to an email address. We create a directed edge between nodes *i* and *j*, if *i* sent at least one message to *j*.

### Baseline algorithms

In order to evaluate the performance of the MATI algorithm, we compare its performance to those of eight baseline algorithms which are described below:

- **Random**: This heuristic chooses *k* random nodes. The behavior depicted in the figures is the average behavior after 100 different random picks.
- **Degree**: A heuristic based on the concept of “degree centrality”, considering high-degree nodes as influential commonly used in sociology literature [43]. This heuristic chooses *k* nodes in decreasing *out-degree* order.
- **Harmonic**: This heuristic is based on the concept of *harmonic centrality* [44] of a node *v*, which can be calculated as follows:
  $$\begin{array}{c} C\left(v\right)=\sum_{u\neq v}\frac{1}{d(u,v)} \end{array}$$
  where *d*(*u*, *v*) indicates the shortest path between the nodes *u* and *v*. The *k* nodes are chosen in decreasing harmonic centrality order.
- **Greedy**: The original greedy algorithm with Monte-Carlo Simulations as described in Section The Influence Maximization (IM) problem. Following the literature [29], we run 10, 000 Monte Carlo (MC) simulations to estimate the spread of any seed set.
- **LDAG**: This algorithm constructs a local direct acyclic graph (DAG) around every node *v* and restricts the calculation of the respective influence inside this local area [24]. The nodes added to the aforementioned DAG of node *v* depend on a threshold *θ*. The individual influence of the nodes added to node *v* has to be larger than this threshold. Here we set *θ* to $\frac{1}{320}$ as used by the authors. The algorithm is tailored for the Linear Threshold diffusion model only.
- **SimPath**: The algorithm computes the influence spread of the nodes by exploring simple paths in their neighborhood [28]. A prunning threshold *η* is used to balance running time and quality of the influence spread of the seed nodes. Here, the pruning threshold is set to 10^−3^ and the look-ahead value *l* is set to 4, as proposed by the authors.
- **IMM**: The **I**nfluence **M**aximization via **M**artingales algorithm adopts a two-phase paradigm which includes *parameter estimation* and *node selection*. In the parameter estimation phase of IMM, the martingales statistical approach is employed to derive the number *θ* of reverse reachable (RR) sets to be chosen before the seed nodes are selected. A (1 − 1/*e* − *ϵ*)-approximation is achieved with *ϵ* being a tunable parameter. In the experiments we use *ϵ* = 0.1. The algorithm is defined for both the Linear Threshold and Independent Cascade models.
- **SKIM**: The **SK**etch-based **I**nfluence **M**aximization (SKIM) algorithm uses per-node summary structures called *combined reachability sketches* that represent the node’s influence coverage across a number *l* of graph instances. The combined reachability sketch is the bottom-*k* min-hash sketch of the combined reachability sketch of the node. Parameter *k* works as a tradeoff between computation and accuracy. In the experiments we use *l* = 64 and *k* = 64. The algorithm is tailored for the Independent Cascade model only.

Unless noted otherwise, the threshold *θ* for the proposed MATI algorithm is set to 0.0001. The value was chosen experimentally, based on performance observation.

### Experimental results

We compare the performance of the aforementioned algorithms, with respect to the quality of seed sets and efficiency aspects.

#### Quality of seed sets

**Fig 4:**
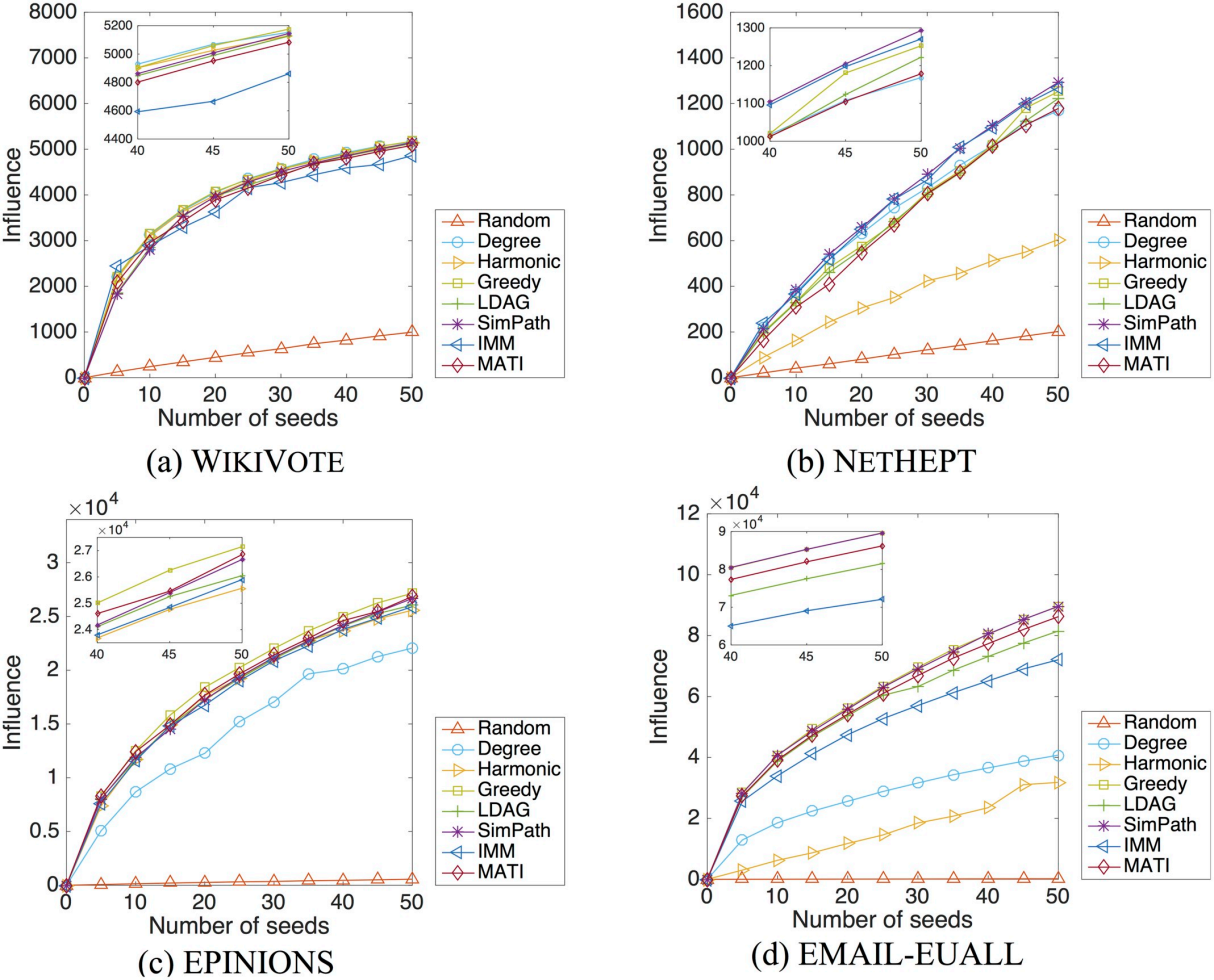
Influence spread in number of nodes for the different algorithms, under the LT model. We show results for the following networks: (a)WikiVote; (b)NetHEPT; (c)Epinions; (d)Email-EuAll. Each plot depicts the influence in number of nodes achieved by the different methods: Random, Degree, Harmonic, Greedy, LDAG, SimPath, IMM and MATI. Each point shows the number of nodes that the respective number of seed nodes—given by the different methods—achieve to influence.

**Fig 5:**
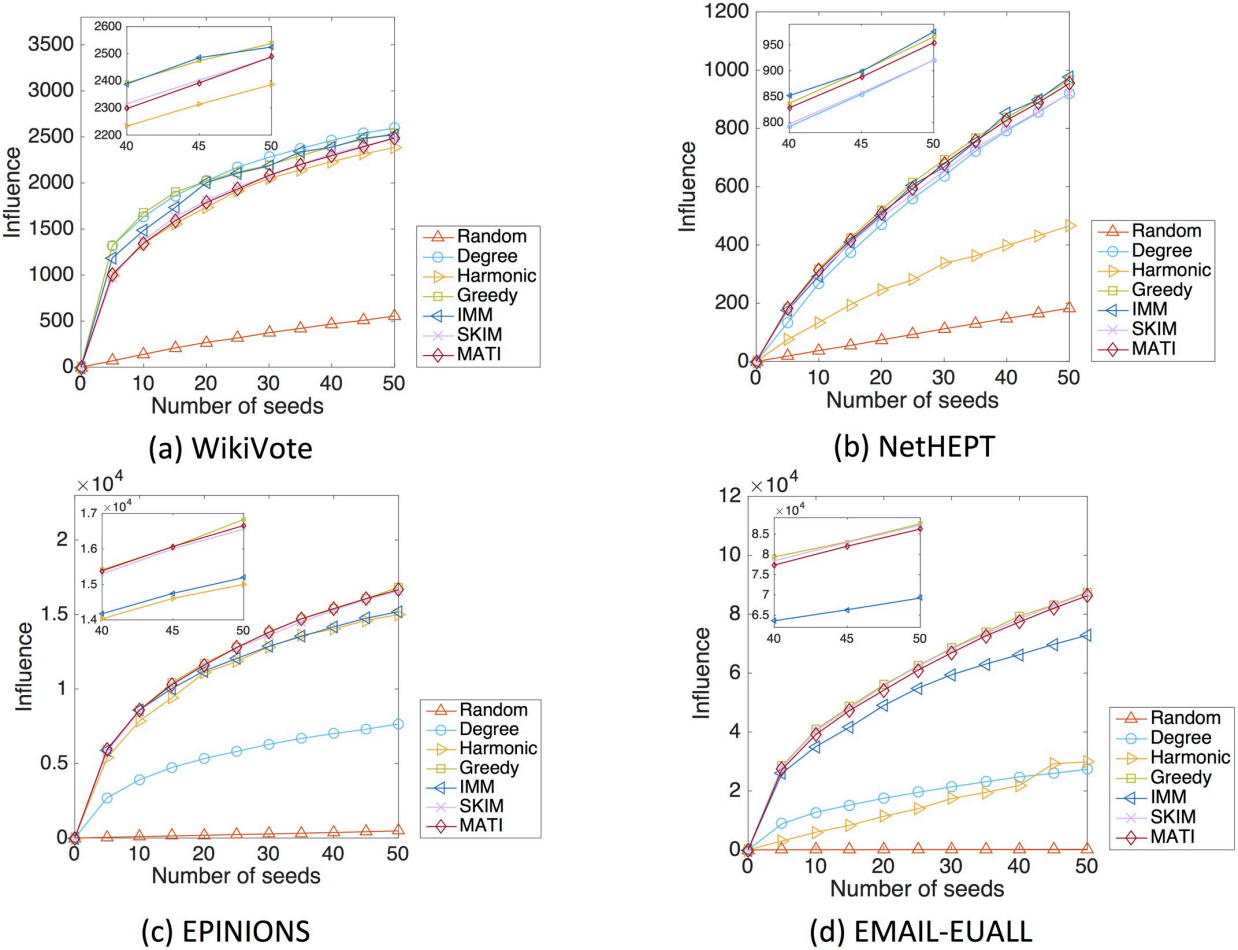
Influence spread in number of nodes for the different algorithms, under the IC model. We show results for the following networks: (a)WikiVote; (b)NetHEPT; (c)Epinions; (d)Email-EuAll. Each plot depicts the influence in number of nodes achieved by the different methods: Random, Degree, Harmonic, Greedy, IMM, SKIM and MATI. Each point shows the number of nodes that the respective number of seed nodes—given by the different methods—achieve to influence.

The quality of the seed sets obtained by different algorithms is evaluated based on the expected spread of influence measured in number of nodes. Figs 4 and 5 show the spread of influence versus the size of seed set, under the LT and IC models respectively.

Under the LT model, the seed sets obtained via MATI are quite competitive in quality compared to those of the Greedy, LDAG, SimPath and SKIM algorithms. For all four datasets, the influence loss for up to 50 seeds is less than 2% with respect to the Greedy algorithm. Under the IC model, our algorithm is still effective, despite the heuristics involved in the influence estimation.

We observe that for both LT and IC models the Random heuristic fails to identify good candidates. On the other hand, the Degree and Harmonic heuristics reveal seed nodes that are actually good candidates for small networks such as WikiVote and NetHEPT but fail to reveal effective seed nodes for larger networks such as Epinions and Email-EuAll. This is due to the fact that the aforementioned heuristics do not take into account that some of the chosen nodes are clustered together and the overlapping influence some nodes occur reduces their final total influence.

We have also performed experiments for different values of the parameter *θ* of our algorithm for the NetHEPT dataset, in order to observe the running time of our algorithm with respect to the influence that is achieved. The results are depicted in Table 3. As *θ* decreases, the running time is always increasing. This is justified by the fact that a smaller *θ* allows computation of influence in a greater neighborhood around each node. In most of the cases, the influence achieved also increases. This can be explained by the fact that the formation of paths of greater length provide a more accurate computation of a node’s influence.

**Table 3:** Comparison of running times in seconds and influence spread in number of nodes for different values of the parameter *θ* under (a) the LT and (b) the IC model for the NetHEPT network.

*θ*	Run. Time (s)	Influence	*θ*	Run. Time (s)	Influence
0.1	1.2	984.7	0.1	1.2	867.77
0.01	6.5	1162.3	0.01	6.8	959.42
0.001	88.6	1209.6	0.001	106.5	938.21
0.0001	708.2	1190.8	0.0001	820.6	950.32
(a)			(b)		

#### Efficiency of MATI

**Fig 6:**
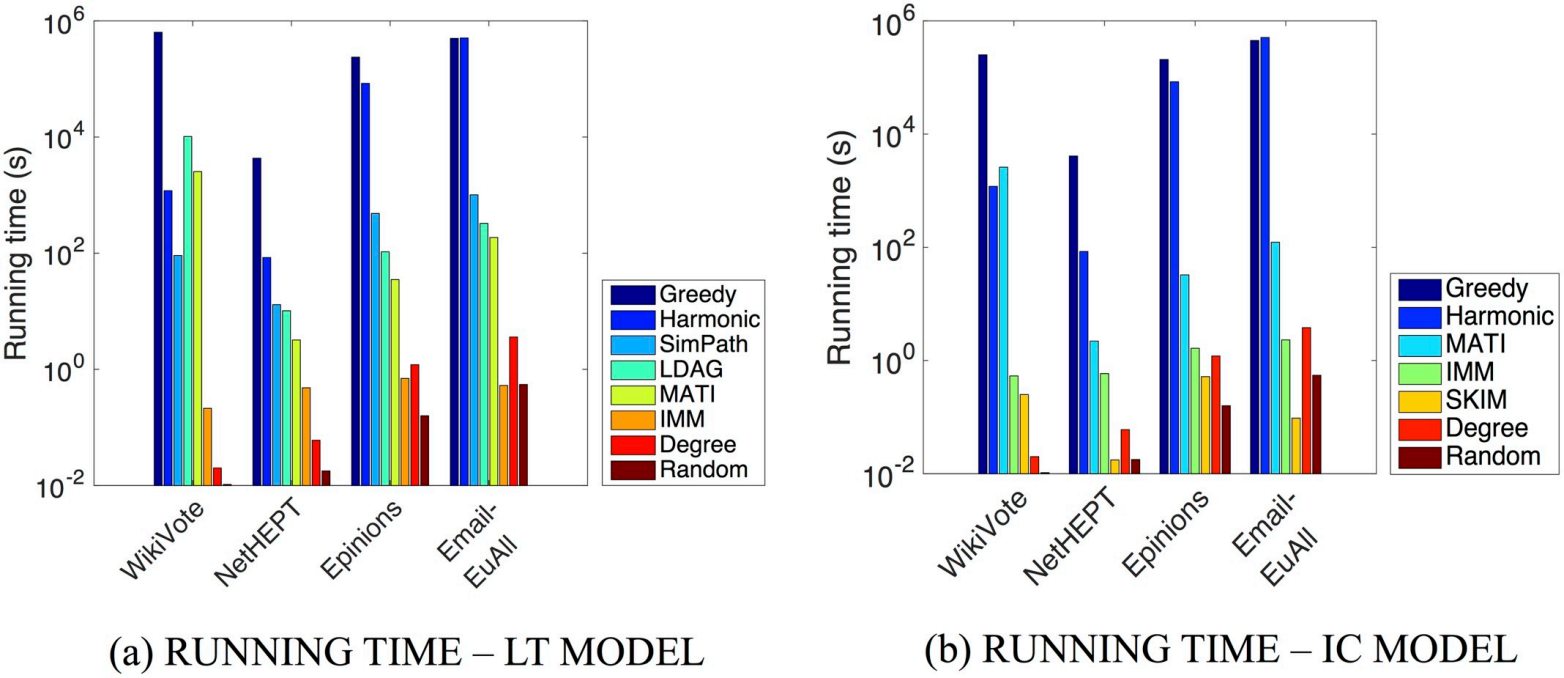
Comparison of running times in seconds of the different algorithms under the (a) LT and (b) IC models. We show results for the following networks: WikiVote; NetHEPT; Epinions; Email-EuAll. Each plot depicts the running time in seconds that each different algorithm requires to produce a group of *k* = 50 seed nodes. Results for the following methods are shown: Random, Harmonic, Degree, Greedy, LDAG, SimPath, IMM, SKIM and MATI.

We have also examined the running time of the proposed algorithm. Fig 6 reports the execution time required by various algorithms for the LT and IC models respectively. The figures have a logarithmic scale on the y-axis. In all cases, MATI is faster than the Greedy and LDAG algorithms. In all datasets except WikiVote, MATI also performs better than SimPath. It takes Greedy more than one week to select 50 seed nodes for datasets such as Epinions. The IMM and SKIM algorithms seem to be slightly faster than our algorithm and this is due to the initial computation of the large data structures described in Section MATrix Influence (MATI) algorithm. The latter computations happen once and despite their “expensiveness” they can facilitate the re-computation of the node’s influence in case there is a node deletion in the network. Nevertheless MATI remains competitive in terms of running time and it will be also competitive in the case of a possible change in the network as mentioned whereas this is not guaranteed for the other baselines. Additionally, MATI’s running time can always be reduced by increasing parameter *θ* which will not result in a significant decrease in the influence spread (see Table 3). It is also worth noticing that even IMM is faster, the influence spread given by the seed nodes proposed is lower than our algorithm’s in some datasets. Specifically we can observe the difference between the influence spread of IMM and MATI in the case of the WikiVote and Email-EuAll datasets for the LT model and that of Epinions and Email-EuAll datasets for the IC model (see Figs 4 and 5 respectively). SKIM is also competitive in terms of running time but is only designed for the Independent Cascade model while MATI is designed for both aforementioned diffusion models. Finally we observe that although the Degree and Random heuristics are time efficient, they fail to output a seed set of high quality.

**Fig 7:**
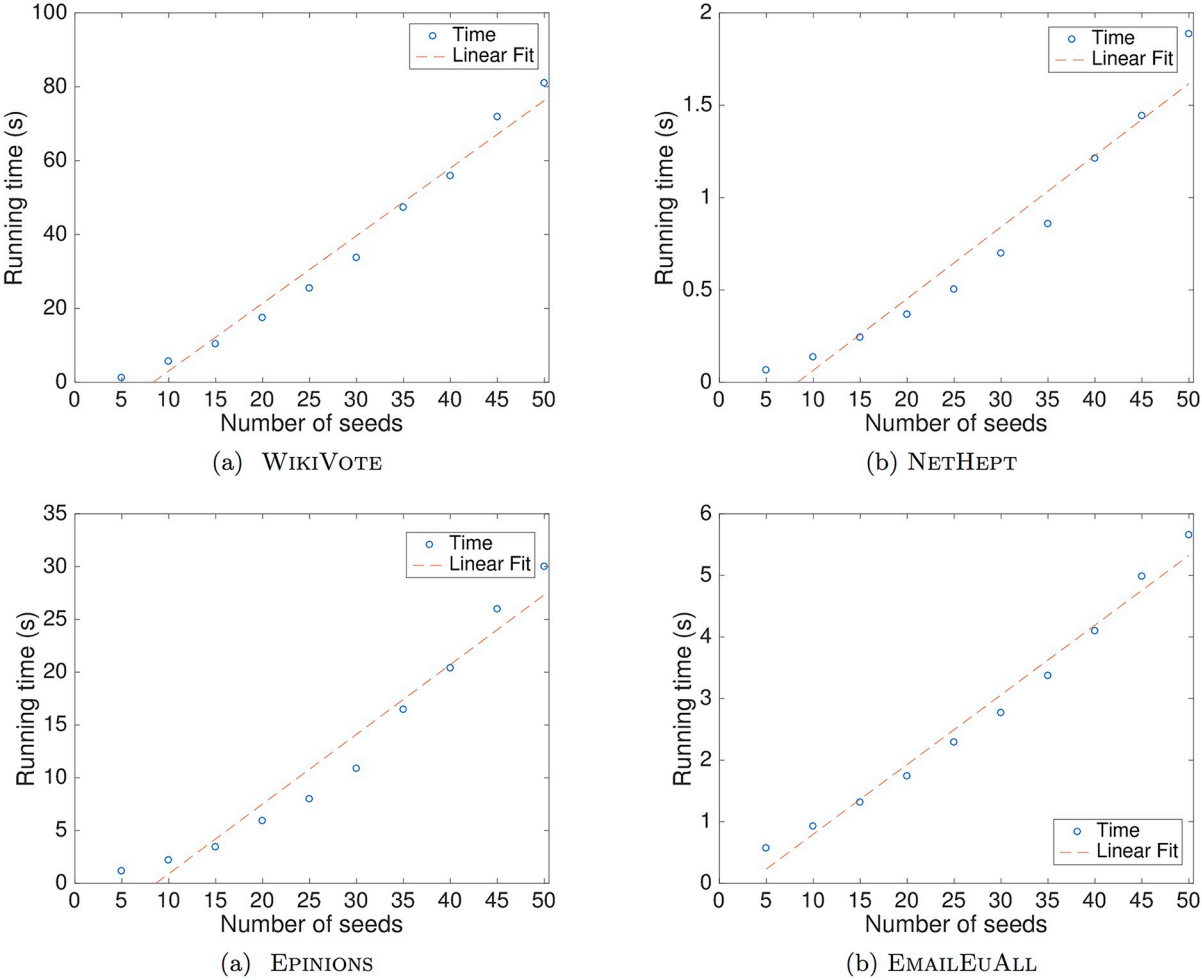
Computation times in seconds of the MATI algorithm for different numbers of seed nodes under the LT model. We show results for the following networks: (a)WikiVote; (b)NetHEPT; (c)Epinions; (d)Email-EuAll. Each plot depicts the running time in seconds that MATI requires to produce a ranging number of seed nodes. We observe that the computation time is close to linear with respect to the number of seed nodes that the algorithm identifies as influential.

**Fig 8:**
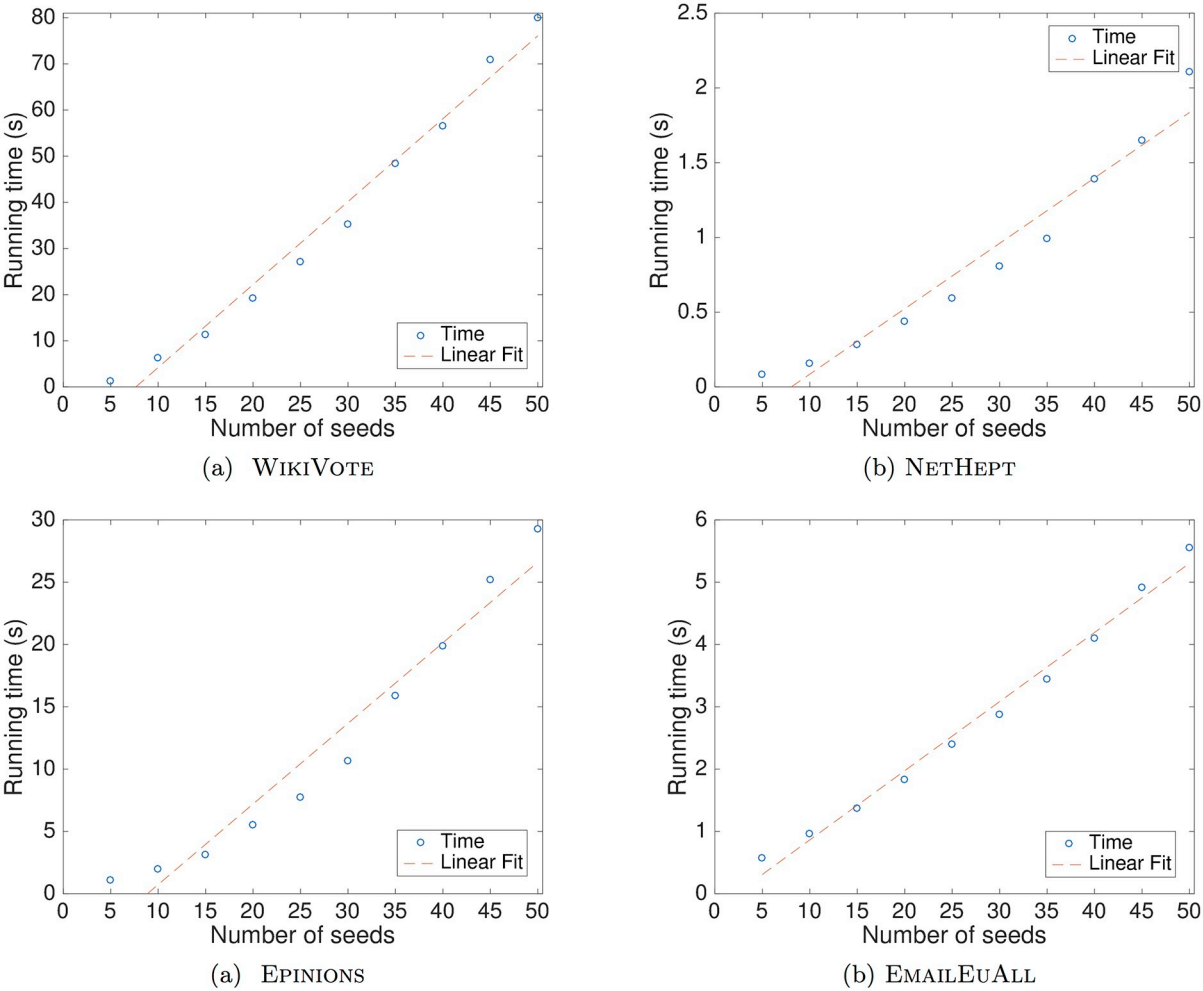
Computation times in seconds of the MATI algorithm for different numbers of seed nodes under the IC model. We show results for the following networks: (a)WikiVote; (b)NetHEPT; (c)Epinions; (d)Email-EuAll. Each plot depicts the running time in seconds that MATI requires to produce a ranging number of seed nodes. We observe that the computation time is close to linear with respect to the number of seed nodes that the algorithm identifies as influential.

In Figs 7 and 8, the running times that the MATI algorithm requires to identify a ranging number of seed nodes are depicted. By using the least squares regression method [45], we observe that the computation time is linear with respect to the number of influential nodes that are identified.

## Conclusion

In this paper we introduced an efficient algorithm for influence maximization in social networks, under both the LT and IC diffusion models. Even though the greedy algorithm has been proven to produce the best results so far in terms of quality of seeds, it does not scale to large networks.

MATI takes into consideration the possible paths that are created in each node’s neighborhood and pre-calculates the nodes’ influences. Our algorithm depends on the creation of large data structures which increases memory consumption, but at the same time facilitates the re-computation of the nodes’ influence in the case of a node or edge deletion in the network. After performing extensive experiments in four datasets, we have shown that MATI is competitive regarding both the quality of seeds and the running time when compared to state-of-the-art algorithms. We plan to further evaluate our algorithm by doing more experiments with larger datasets and comparing it with more baseline methods. Additionally, we will be experimenting with heuristics that can further speed up the running time and efficient data structures that may reduce the memory consumption of our algorithm. Finally, we will be studying how our method can be extended for the case of a dynamic graph where nodes and edges are added or deleted from the network.
